# Voyage D'Études Médicales

**Published:** 1907-07-13

**Authors:** Leonard Williams

**Affiliations:** 8 York Street, Portman Square, W.


*To the Editor of* THE HOSPITAL.

### VOYAGE D'ÉTUDES MÉDICALES.

*To the Editor of* THE HOSPITAL.

SIR,—May I call the attention of your readers to the facilities for seeing certain of the French health-resorts which are annually offered by the organisation which bears the above title. For the purposes of these visits France is divided into five districts, each of which contains a large number of spas and mineral-water stations. Every year one of these districts is made the object of a carefully arranged inspection. The time chosen is the first fortnight in September. The party, which is strictly limited to 100 persons, travels under the most favourable conditions. A first class special train conveys the members from place to place. The hotel accommodation and the food is provided for in advance. The care and transport of the luggage is undertaken by the organisers in such a way that a member's valise left at the proper hour outside his bedroom at the place of departure, is found inside his bedroom at the next halting-place. The advantages of the particular stations visited are explained at each place by Professor Landouzy, who acts as the president of the company. This year the health-resorts to be visited are those in the district of the Vosges, which include such well-known places as Contrexéville, Vittel, Martigny, Plombières, Luxueil, and many others. The *rendezvous* is at Reims on August 31, and the company parts at Divonne, on the lake of Geneva, on September 13. Having now taken part in five of these trips, I can assure your readers that there is no more agreeable or instructive manner of spending a part of one's summer holiday. The price (300 francs or £12) is astonishingly low, especially when it is realised that this includes everything from the *rendezvous* to the *dislocation*. There are no tips or extras of any kind. Medical men and their wives, and medical students, are allowed to join. Those coming from this country are always sure of a particularly warm welcome.

I shall be happy to supplement this information if any of your readers should desire further particulars on the subject.

Yours faithfully,

LEONARD WILLIAMS.

8 York Street, Portman Square, W.

